# Steroid-Induced Diabetes: Is It Just Unmasking of Type 2 Diabetes?

**DOI:** 10.5402/2012/910905

**Published:** 2012-07-05

**Authors:** Lisa R. Simmons, Lynda Molyneaux, Dennis K. Yue, Elizabeth L. Chua

**Affiliations:** ^1^Department of Endocrinology, Diabetes Centre, Royal Prince Alfred Hospital, Level 6, West Wing, Camperdown, NSW 2050, Australia; ^2^Sydney Medical School, The University of Sydney, D06, Sydney, NSW 2006, Australia

## Abstract

*Aims*. We compared the demographic profile and clinical characteristics of individuals with new onset steroid-induced diabetes (NOSID) to Type 2 diabetes (T2DM) patients with and without steroid treatment. *Methods*. The demographic profile and clinical characteristics of 60 individuals who developed NOSID were examined and matched to 60 type 2 diabetes patients receiving steroid therapy (T2DM+S) and 360 diabetic patients not on steroids (T2DM) for age, duration of diabetes, HbA1c, gender, and ethnicity. *Results*. Patients who developed NOSID had less family history of diabetes (*P* ≤ 0.05) and were less overweight (*P* ≤ 0.02). NOSID was more commonly treated with insulin. Despite a matching duration of diabetes and glycaemic control, significantly less retinopathy was found in the group of patients with NOSID (*P* < 0.03). *Conclusions*. It appears that steroid treatment primarily precipitated diabetes in a group of individuals otherwise less affected by risk factors of diabetes at that point in time, rather than just opportunistically unmasking preexisting diabetes. Furthermore, the absence of retinopathy suggests that patients with NOSID had not been exposed to long periods of hyperglycaemia. However, the impact of the underlying conditions necessitating steroid treatment and concomitant medications such as immunosuppressants on diabetes development remain to be defined.

## 1. Introduction

Glucocorticoids are commonly used to treat a wide variety of both acute and chronic illnesses. Their use can be accompanied by a multitude of side effects, including hyperglycaemia and can worsen preexisting diabetes or precipitate new “steroid-induced” diabetes [1–5].The term “steroid diabetes” was coined by Ingle in the 1940s to describe the hyperglycaemia noted in rats receiving glucocorticoids [6–8]. Similarly, glucocorticoid-induced hyperglycaemia has long been noted in humans [9–11]. Steroids elevate blood glucose levels by increasing hepatic glucose production and inhibiting glucose uptake into muscles. They also have a complex effect on beta cell function [9–11]. Yet, the pathophysiology of glucocorticoid-induced diabetes and the relative contributions of both *β*-cell dysfunction and insulin resistance are still to be elucidated [4, 5, 7, 12]. In addition, the clinical course and optimal treatment of New-Onset Steroid Induced Diabetes (NOSID) still remain unclear.

Hyperglycaemia can be one of the troubling consequences of both short- and long-term steroid use. NOSID has been identified in between 1.5% and 47% of patients. Variability of this incidence is thought to be due to differences in patient population, treatment protocols, and the definition of diabetes [13–16]. Despite being well established that steroids have a pronounced hyperglycaemic effect [17], the risk factors and impact of developing NOSID are poorly characterised or quantified. Review of the literature suggests that increasing dose, duration of therapy, ethnicity, age, underlying disease, and BMI may be risk factors for the induction of NOSID [18–22]. Studies of renal transplant recipients have shown that the incidence of NOSID increases with steroid dose and is often first detected after increasing the steroid dose during episodes of rejection [23]. The association of traditional risk factors such as obesity and family history with the development of hyperglycaemia remains unclear [24–28]. The impact of NOSID on diabetic complications has also not been well characterized.

The aim of our study is to examine the demographic profile and clinical characteristics of patients who develop NOSID. We compared the profile of individuals with NOSID to type 2 diabetes (T2DM) patients with and without steroid treatment in an attempt to determine their similarities and differences, both in diabetogenic risk factors and diabetic complications.

## 2. Materials and Methods

Data from individuals attending the Royal Prince Alfred Hospital Diabetes Centre in Sydney during a 10-year period were collected and made available for review. NOSID was defined as diabetes diagnosed for the first time during steroid therapy. The criteria used were more than one random BGL >11 mmol/L or more than one fasting BGL >7.0 mmol/L following the initiation of steroid therapy [29]. Each patient was assessed for the presence of diabetic complications using a standardised protocol described previously [30]. Information collected at these visits includes demographic details, past medical history, weight, HbA1c, albuminuria, blood pressure, and fasting lipids. Nephropathy was evaluated by way of creatinine and the urine albumin to creatinine ratio. Measurement of vibration perception threshold by biothesiometer, sensation via monofilament, and ankle reflexes allowed evaluation for signs of neuropathy. In addition, retinopathy was assessed by direct fundoscopy through dilated pupils or via a report from the individual's treating ophthalmologist.

Complete complications assessment data were available for a total of 60 patients with NOSID. Data from these individuals were compared with complications assessment data for patients with type 2 diabetes as well as those with type 2 diabetes receiving steroids at the time of complications assessment. Patients with NOSID were matched 1 : 1 with patients known to have type 2 diabetes receiving steroid therapy (T2DM+S) and 1 : 6 with those not on steroids (T2DM). Matching criteria included age, gender, ethnicity, duration of diabetes, and HbA1c. A total of 480 patients were studied made up of 60 individuals diagnosed with NOSID, 60 type 2 diabetics receiving steroid therapy, and 360 patients with type 2 diabetes not on steroids.

The metabolic syndrome was defined as a score of 3 or greater according to the World Health Organization 1999 criteria [31, 32]. As all participants fulfilled the criteria for hyperglycaemia, at least two of remaining criteria were required.

Data were analysed using NCSS (Number Cruncher Statistical System, Kaysville, UT) 2007. Data were grouped into three groups: (1) new-onset steroid-induced diabetes, (2) type 2 diabetes on steroids, and (3) type 2 diabetes. Optimal data matching procedures were used to match the three groups. Potential bias was avoided by matching the patients for confounders that may influence complications status. The data were then matched by age, duration of diabetes, HbA1c, ethnicity, and gender. A propensity score was calculated for the matching procedure using logistic regression, all confounders were included in the model. Mahalanobis Distance within Propensity Score Calipers (no matches outside calipers) were used to calculate the distance between the three groups. Patients were matched 1 : 1 with type 2 diabetics receiving steroid therapy and 1 : 6 with type 2 diabetics not on steroids. Continuous data were checked for normality and presented as mean and standard deviation or median and interquartile range. ANOVA was used to compare means or medians of the three groups. Bonferroni or Kruskal-Wallis adjustments were performed between the three groups to adjust for multiple groups. Categorical data was presented as percentage. Chi-square and Fisher's exact tests were used to compare the groups. Statistical significance was accepted at *P* < 0.05.

## 3. Results

A total of 271 individuals with NOSID attended the Diabetes Centre for assessment and treatment. Patients were treated with oral steroids at a dose which ranged between 5 mg and 40 mg daily. Sixty of these individuals with complete demographic data and complications assessment were included in this study and had their data analysed. The most common reason for requiring steroid therapy and thus needing review at our Diabetes Centre was posttransplant immunosuppression (75%). Other conditions included respiratory, renal and rheumatological disease (25%). The demographic and clinical profiles of study patients are shown in Table 1.

Those individuals who developed NOSID were noted to have less family history of diabetes when compared with patients with Type 2 diabetes receiving steroid therapy and type 2 diabetes alone (*P* ≤ 0.05). Patients who developed NOSID weighed less (*P* ≤ 0.02) than those with known type 2 diabetes, despite the steroid therapy which is known to cause weight gain. There was, however, no significant difference (*P* = 0.5) in the prevalence of metabolic syndrome between the three groups.

Overall, NOSID was more commonly treated with insulin (*P* < 0.0001).

Despite a matching duration of diabetes, significantly less retinopathy was found in the group of patients with NOSID (*P* < 0.03). Interestingly, none of the patients who developed NOSID were found to have retinopathy.

Macrovascular complications were significantly lower (*P* < 0.006) in the NOSID group when compared with type 2 diabetics receiving steroid therapy. This may be due to the fact that patients with type 2 diabetes also receiving steroids were likely to be more unwell and have more active comorbidities than those patients on steroids alone. Although a difference exists between the patients with NOSID and those known to have type 2 diabetes alone, this result does not reach statistical significance (*P* = 0.4).

## 4. Discussion

Steroids exert a variety of changes that lead to hyperglycaemia or exacerbate preexisting diabetes. The causative mechanisms of hyperglycaemia are multifactorial and so too are the clinical characteristics and demographics of individuals likely to develop NOSID [13, 17, 24–28, 33].

It is known that being overweight is often associated with impaired glucose tolerance and increased risk of developing type 2 diabetes [21, 34, 35]. This study shows that the group of patients who developed diabetes following steroid therapy not only weighed less, when compared to individuals with type 2 diabetes receiving or not receiving steroids, but that despite treatment with steroids, which in itself may cause weight gain, obesity was not a distinctive feature. Furthermore, type 2 diabetes is typically associated with a strong family history [36–39]. By contrast, patients with NOSID in this study had less family history of diabetes. If NOSID was simply type 2 diabetes uncovered opportunistically (due to concurrent illness or steroid treatment), a similar prevalence of family history and obesity would be expected in all groups. These two findings are more consistent with the notion that patients with NOSID have less risk factors for diabetes. They only become diabetic under the stress of steroid treatment.

In our cohort of patients who developed steroid-induced diabetes, none of the individuals developed diabetic retinopathy despite the same duration of disease and HbA1c as patients with known type 2 diabetes. As duration of chronic hyperglycaemia is the fundamental prerequisite for diabetic retinopathy in patients with both type 1 and type 2 diabetes [40–47], the individuals with NOSID are likely not to be hyperglycaemic sufficiently long enough to develop end-organ damage [46–52]. The early detection and intensive glycaemic control in this cohort of patients may have prevented or delayed the onset and progression of diabetic retinopathy. Our observation has practical clinical significance. If a patient with NOSID has no diabetic retinopathy at the time of diagnosis, routine screening for this microvascular complication is not a high priority for the first few years. In view of the high and increasing demand for diabetes services, this would help to prioritise treatment.

The underlying disease for which steroid therapy is instituted is often inflammatory or cytokine mediated which may in its own right affect a patient's glycaemic control. Furthermore, a large proportion of our study population was receiving steroid therapy after transplantation. The impact of steroid use in these individuals is confounded by the use of calcineurin inhibitors (particularly tacrolimus) and sirolimus which contribute to the risk of glucose intolerance possibly by suppressing insulin production [13–16, 23–26]. Additional confounding variables that may affect the incidence and cause of steroid-induced diabetes in the cohort who underwent organ transplantation include the transplant itself, hepatitis C virus status befor transplant, and the patient's weight prior to transplantation [13, 18, 26]. In addition, in patients undergoing renal transplantation, pretransplant diabetes may be masked by diminished insulin metabolism associated with kidney dysfunction [13, 25, 26]. The influence of these factors require further investigation.

Our study was based on a modest sample size; however the subjects in all three groups were well matched for age, duration of diabetes, HbA1c, gender, and ethnicity. Although, retrospective in analysis, the data on clinical parameters and complications was collected prospectively in the same way for all three groups, according to a standardised clinical protocol [30]. A long-term prospective study to confirm the risk factors, demographic profile, and clinical characteristics that predispose individuals to developing steroid-induced hyperglycaemia will provide further insight into the underlying characteristics and profile of individuals more susceptible to NOSID.

## Figures and Tables

**Table 1:** Demographic and clinical profile of patients in the three matched groups.

	NOSID (*N* = 60)	T2DM+S (*N* = 60)	T2DM (*N* = 360)	
Age (yrs)	59.2 ± 11.5	59.8 ± 9.8	59.3 ± 10.5	
Duration DM (yrs)	4.5 [1.6–7.7]	5.0 [2.1–7.5]	4.2 [0.8–8.1]	
Males (%)	52	52	52	
Anglo-Celtic (%)	42	42	42	
HbA1c (%)	6.5 ± 1.2	6.6 ± 1.2	6.6 ± 1.2	
Family history (%)	35*	59	62	*P* < 0.05
Weight (kg)	75.8 ± 18.7*	84.9 ± 16.4	82.8 ± 19.6	*P* = 0.02
Diabetes treatment (%)				*P* < 0.0001
Diet	17	20	18	
Oral Hypoglycemics	38	40	72	
Insulin	45	40	10	
Retinopathy (%)	0^∗^	10	7	*P* < 0.03
Macrovascular (%)	10^ ¶^	30	14	^ ¶^ *P* = 0.006
Metabolic syndrome (%)	57	70	63	*P* = 0.5

Different from ^∗^both groups or ^¶^T2DM+S.

Data are presented as mean, median, and interquartile range.
